# Dysregulation of the hypothalamus-pituitary-adrenal axis predicts some aspects of the behavioral response to chronic fluoxetine: association with hippocampal cell proliferation

**DOI:** 10.3389/fnbeh.2014.00340

**Published:** 2014-09-30

**Authors:** Wahid Khemissi, Rai Khalid Farooq, Anne-Marie Le Guisquet, Mohsen Sakly, Catherine Belzung

**Affiliations:** ^1^Inserm U930 Eq 4, UFR Sciences et TechniquesTours, France; ^2^Université François RabelaisTours, France; ^3^Laboratoire de Physiologie Intégrée, Faculté des Sciences de Bizerte, Université de CarthageBizerte, Tunisia

**Keywords:** antidepressant resistance, fluoxetine, corticosterone, hippocampal neurogenesis, unpredictable chronic mild stress, dexamethazone suppression

## Abstract

In depressed patients, antidepressant resistance has been associated with dysregulation of the hypothalamus-pituitary-adrenal (HPA) axis but the underlying mechanisms are poorly understood. The scope of this study was to try to create HPA-related antidepressant resistance in mice and to investigate adult hippocampal neurogenesis as a putative mechanism of antidepressant resistance. Mice were subjected to a 9 week Unpredictable Chronic Mild Stress (UCMS). After a 2 weeks drug-free period, mice were segregated in two groups, according to the percentage of corticosterone suppression after dexamethasone injection: High suppression (HS) and Low suppression (LS) mice. From the 5th week onwards, fluoxetine at a dose of 15 mg/kg (i.p.) was administered daily and at the end of 8th week, a battery of behavioral tests assessing the emotional, cognitive, and motor aspects of UCMS-induced depressive-like behavior was applied. Results show that fluoxetine-induced antidepressant effects were observed with higher amplitude in HS when compared to LS on various behavioral phenotypes, like coat state, novelty suppression of feeding, splash test and nest test. The same profile was found concerning the immunohistochimical analysis of ki-67 positive cells in the dentate gyrus of the hippocampus, which is a marker of neuronal proliferation, but not for doublecortin labeling. This suggests that the failure of fluoxetine to induce antidepressant effects may be associated to the poor ability of the compound to stimulate cell proliferation in the hippocampus.

## Introduction

Major depressive disorder, the most common psychiatric disorder, is estimated to affect around 120 million people in the world (Hashimoto, 2011), with an estimated lifetime prevalence of about 17% in the USA (Kessler et al., 2005). Depressive disorder is characterized by depressed mood, anhedonia and difficulties in concentration (American Psychiatric Association, 1994; Perahia et al., 2009; Willner et al., 2013). Selective serotonin reuptake inhibitors (SSRIs) such as fluoxetine are the most common form of medication to treat major depression. However, approximately 50% of patients do not respond adequately to treatment with conventional antidepressants (Trivedi et al., 2006; Fava et al., 2008). Resistance to antidepressants is related to several factors like the dysregulation of the hypothalamus-pituitary-adrenal (HPA) axis (Belzung and Billette de Villemeur, 2010) or reduced hippocampal adult neurogenesis (Santarelli et al., 2003; El-Hage et al., 2013). Recently, we have shown that newborn hippocampal neurons are necessary to enable chronic antidepressants to counteract stress-induced HPA dysfunction (Surget et al., 2011). However, there are no reliable data enabling to link HPA-related antidepressant resistance to alterations in hippocampal cell proliferation or neurogenesis, which may be due to lack of validated animal models.

The unpredictable chronic mild stress (UCMS) model of depression has been widely used in antidepressant screening (Garcia et al., 2010; Nollet et al., 2013). This model was developed in order to mimic a variety of neurochemical and behavioral alterations similar to the ones seen in the human depressive disorder (Jindal et al., 2013; Nollet et al., 2013). It has been also shown to elicit HPA axis dysregulation (Surget et al., 2011) similar to the one seen in depressed subjects (Heim et al., 2000; Belzung and Billette de Villemeur, 2010) as well as decreased hippocampal neurogenesis (Surget et al., 2008, 2011).

The objective of the present study was (1) to design an animal model of HPA-related antidepressant resistance; and (2) to use this model to investigate the relationship between HPA-dysregulation and adult hippocampal cell proliferation and neurogenesis.

## Materials and methods

### Animals

Male BALB/cByJ mice aged 7 weeks (*n* = 52) at their arrival in the laboratory were used. All mice were acquired from the Centre d’Elevage Janvier (Le Genest Saint Isle, France). After arrival at the lab, animals were group-housed (4–5 per cage) and kept under standard conditions (12/12 h light-dark cycle with lights on at 9:00, off at 21:00, room temperature: 22° ± 1°C, food and water *ad libitum*) in standard cages (42 cm × 27 cm × 16 cm) with a shelter and a tube for 1 week prior to the start of the experiment. All animal’s care and treatment were in accordance with the European Community Council directive 86/609/EEC.

### Unpredictable Chronic Mild Stress (UCMS)

**Figure 1:**
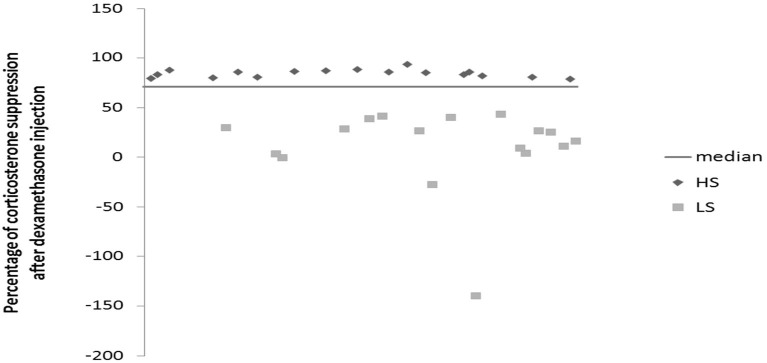
**Effects of 2 weeks of unpredictable chronic mild stress (UCMS) on the dexamethasone suppression**. Data represent the percentage of corticosterone suppression after dexamethasone injection HS (*N* = 17), LS (*N* = 17).

Mice were daily subjected to various stressors usually in the morning and in the afternoon according a semi-random schedule for 9 weeks. 1 week after their arrival at the lab, mice were isolated in individual cages (24 cm × 11 cm × 12 cm) in order to prevent them of injuries related to UCMS-related agression. The stressors used consisted of dampened sawdust, substitution of sawdust with water at 21°C, removal of sawdust, tilting the cage by 45°C, restraint stress, repeated changes of sawdust, alterations of the light and dark cycle, placing a mousse into a cage that has been occupied by another mouse and predator sounds (Surget and Belzung, 2008). After 2 weeks of stress exposure, we measured the regulation of the HPA axis using the dexamethasone suppression test. Median of dexamethasone-induced suppression was 71.54%. According to the percentage of corticosterone suppression after dexamethasone injection, we divided the mice into two groups (Figure 1): High suppression (HS) and Low suppression (LS) according to the median. In order to better segregate both groups, the animals whose dexamethasone suppression score was 10% higher or lower than the median (71.54 +/− 7.15) were excluded, which corresponded to 18 mice. Therefore, final number of mice was *n* = 34 (17 HS and 17 LS). From the 5th week onwards, we administered either vehicle or fluoxetine at 15 mg/kg (ip) daily. At the end of 8th week, we applied behavioral tests. Four mice died before the behavioral testing occurred. Mice were sacrificed after 9 weeks of exposure to UCMS.

### Dexamethasone suppression test

**Figure 2:**
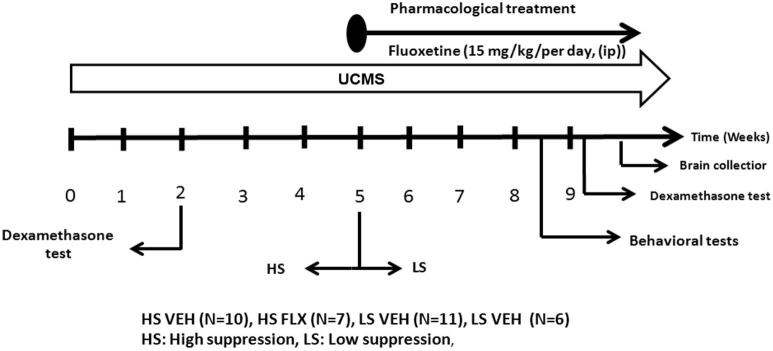
**The experimental protocol**. All mice were subjected to a 9 week UCMS. After 2 weeks of UCMS, a dexamethasone suppression test was performed. 3 weeks later, mice were divided in two cohorts according to their level of corticosterone suppression. The UCMS procedure continued 5 further weeks. In each cohort, half of the mice were treated daily with vehicle while the other half was daily treated with fluoxetine (ip, 15 mg/kg/per day). At the end, behavioral tests were performed as well as a second dexamethasone suppression test. HS: High suppresser, LS: Low suppresser, VEH: vehicle, FLX: fluoxetine.

The dexamethasone suppression test was done after 2 weeks of a drug-free UCMS as well as at the end of the 9 weeks. Dexamethasone has been extensively used to probe HPA axis feedback. Mice were injected with Dexamethasone-Phosphate (D-1759, Sigma-Aldrich) 0.1 mg/kg or vehicle (0.9% NaCl) intraperitoneally (ip) on two separate occasions: if mice were treated with vehicle at the first occasion, after 2 days the same mice were administered dexamethasone in a counterbalanced way, so that half mice in each experimental group were treated first with vehicle and the other half first with dexamethasone. 30 min after these injections, the mice were subjected to open field exploration for 5 min. Blood was collected from submandibular region (submandibular bleeding targets the point where the orbital veins join the submandibular vein to form the jugular vein) 2 h after each injection in EDTA added cones. Blood was centrifuged at 5000 rpm for 10 min and plasma was pipetted for conservation at −80°C. Corticosterone dosage was done by radioimmunoassay using corticosterone 125Ikit (MP Biomedicals, New York, NY) according to the manufacturer’s protocol. Radioactivity was calculated using gamma counter. The percentage of suppression was calculated as following: (the value of vehicle—the value of dexamethasone/the value of vehicle)*100. The dexamethasone test was done after 2 weeks of UCMS and a second time at the end of the UCMS protocol (Figure 2). Regarding the dosage at the end of the UCMS, the data of seven mice are missing due to technical problems.

### Drugs

The SSRI fluoxetine hydrochloride (Sequoia Research Products Pangbourne, UK) was prepared in saline (0.9% sodium chloride). Fluoxetine (15 mg/kg/day) and vehicle were administered ip daily from the 5th week of the UCMS procedure until the sacrifice.

### Coat state

The coat state of each animal was measured weekly as one of the markers of UCMS-induced depressive-like behavior. Mice exposed to UCMS display a progressive degradation of their coat state. It was measured on seven body parts of the mouse: head, neck, dorsal coat, ventral coat, tail, forepaws and hind paws. For each body part, a score 0 was given for good state (smooth fur), a score 0.5 was given for moderate degradation (fur with some spiky patches) and a score 1 for unkempt coat (bad fur). The total score given for coat state is the sum of scores obtained from the seven body parts.

### Novelty Suppression of Feeding (NSF)

The testing apparatus consisted of a wooden arena (30 × 30 × 20 cm) with the floor covered by 2 cm of sawdust (Surget et al., 2008). Testing was done under red light. The animals were food-deprived 12 h before the test. At the time of testing, a single pellet of food was placed on a white paper in the center of arena. The animal was then placed in a corner of the arena with the head facing the wall. The latency to start consuming the pellet was recorded during a 3 min period as well as the frequency of sniffing. To control for feeding drive or appetite, animals were immediately returned to their home cage after the test and the amount of food consumed during the subsequent 5 min was measured (home food consumption).

### Splash test

A sucrose solution was squirted onto the dorsal coat of the animals in their home cage and the duration and the frequency of grooming was recorded during the 5 min following the vaporization (Ducottet and Belzung, 2004).

### Actograph

Home cage activity was recorded for 2 h using a photoelectric actograph. All stressed mice were isolated 24 h before the test to avoid activity induced by a novel environment. Home cages were placed in the device which consisted of a 20 × 20 cm square plane with photobeam detectors. The plane movements were automatically detected as the animals crossed through the detectors and a score was established.

### Nest test

Mice were isolated in individual cage (22 cm × 16.5 cm × 14 cm), after one Nestlet was introduced in each cage (3 g per cage). The quality of the nest was scored 24 h after introduction of the nest building material in the cage (Deacon, 2006; Nollet et al., 2013).

### Tail suspension test

Mice were suspended by the tail using adhesive tape to a rod 60 cm above the floor. The trials were conducted for a period of 6 min and were video recorded. The behavioral measure was the duration of immobility, interpreted as behavioral despair.

### Sacrifice and immunochemistry

Intracardiac perfusions were performed in order to investigate cell proliferation and adult neurogenesis in the dentate gyrus of the hippocampus. Mice were deeply anesthetized (sodium pentobarbital 40 mg/kg i.p) and perfused through the heart with 80 ml of saline followed by 200 ml of 4% paraformaldehyde in 0.1 M PBS (ph = 7.4). Brains were removed and post fixed in the same fixative and cryoprotected in a 20% sucrose solution overnight at 4°C before being processed. Coronal sections (40 µm thickness) were cut in cryostat (Leica CM 30505) and divided in four sections series allowing different immunohistochimical procedures. Free floating sections were washed in 0.1 MPB, 50% of ethanol and 3% H_2_O_2_. Sections were incubated at room temperature in a rabbit anti ki-67 antibody (Abcam, ab15580, 1:1000) followed by three washes in 0.1 M PBS and a 2 h-incubation in secondary donkey anti-rabbit IgG biotynilated antibody (Jackson immuno Research diluted 1:500). Then sections were incubated in avidin-biotin-peroxidase complex (Vectastain ABC Kit, Victor Laboratories Burlingame, CA, USA, diluted 1:100) for 1 h and reacted with freshly prepared diaminobenzidine-HCL (DAB, Sigma—Aldrich D4293) without metal enhancement (Sigma-Aldrich, D4293). The section were then rinsed in PB, mounted on gelatinized glass slides, dehydrated, cleared in claral and cover slipped with a Eukitt. All sections were counterstained with crezyl violet (Santa Cruz, SC-214775). The same procedures were used for doublecortin positive cells using goat anti DCX as a primary antibody (Santa Cruz, SC-8066, 1:500) and anti goat (A-11056, 1:500) as a secondary antibody.

### Data analysis

Cell proliferation and neurogenesis were examined in the septal (bregma −0.94 to −1.58 mm), intermediate (bregma −1.70 to −3.28 mm) and temporal parts (bregma −3.40 to −3.88) of the hippocampus (Franklin and Paxinos, 2008). The granule cell layer surfaces were determined with Axiovision software from pictures obtained at x10 objective lens at corresponding levels on crezyl violet staining to express numbers of immunoreactive cells per square millimeter of respective areas and finally per cubic millimeter by multiplying by the thickness of sections (40 µm). All quantifications were performed by an investigator blind to stress and treatment. All sections in each part of the hippocampus were examined with a Leica DM 2000 microscope.

### Statistical analysis

For behavioral tests, dexamethasone suppression and histological analysis, group comparisons were performed using non-parametric-tests as data did not follow a normal distribution and variance were not homogeneous. The Kruskall-Wallis test was performed followed by a Mann Whitney *U*-test including corrections for multiple comparisons when required. For comparisons between vehicle and dexamethasone injection in a given experimental group, we used Wilcoxon test. All data are expressed as the mean ± SEM.

## Results

### Effects of fluoxetine on coat state and on behavioral tests

**Figure 3:**
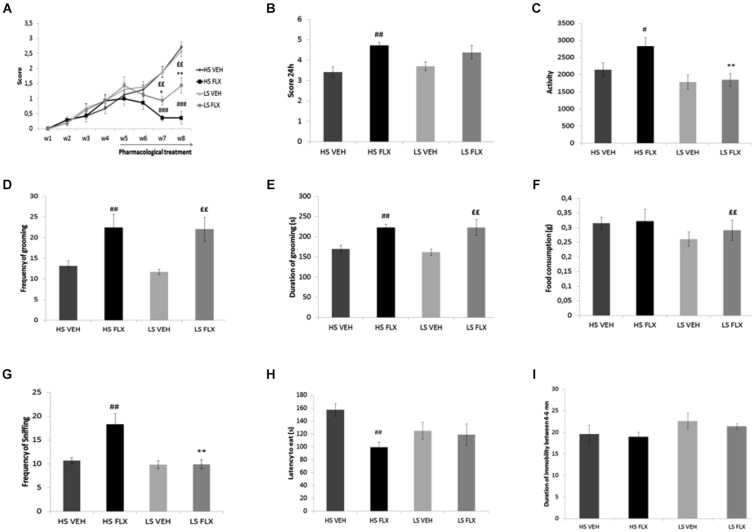
**Effects of UCMS and 4 weeks fluoxetine treatment (15 mg/kg/per day) (FLX) or Vehicle (VEH) on the behavior in High suppresser (HS) and Low suppresser (LS) mice (HS VEH (*n* = 10), HS FLX (*n* = 7), LS VEH (*n* = 11), LS FLX (*n* = 6)**. **(A)** Coat state **(B)** Nest test **(C)** Locomotor activity. **(D)** Frequency of grooming in the splash test. **(E)** Duration of gooming in splash test. **(F)** Food consumption in novelty suppression of feeding. **(G)** Frequency of sniffing in novelty suppression of feeding. **(H)** Latency to eat in the novelty suppression of feeding test. **(I)** Immobility in the tail suspension test. HS FLX vs. LS FLX: * *p* < 0.05, ** *p* < 0.01; HS FLX vs. HS VEH: ^#^ *p* < 0.05, ^##^ *p* < 0.01, ^###^ *p* < 0.001; LS FLX vs. LS VEH: ^££^*p* < 0.01.

As seen from Figure 3A, Kruskall Wallis test revealed overall differences in the coat state after 7th and 8th weeks UCMS (respectively *H*_(3,34)_ = 22.35, *p* < 0.001; *H*_(3,34)_ = 20.34, *p* < 0.001). The results show that the UCMS-induced coat state degradation was reversed by fluoxetine in both HS and LS. Mann Whitney comparisons showed significant difference between HS FLX vs. HS VEH (*p* < 0.01) (week 7 and week 8) and LS VEH vs. LS FLX (*p* < 0.01) (week 7 and week 8). Further, significant difference was found between LS FLX vs. HS FLX for week 7 (*p* < 0.05) and for week 8 (*p* < 0.01). No significant difference was found between HS VEH vs. LS VEH.

Kruskall Wallis test revealed differences among groups in the nest building score (*H*_(3,34)_ = 11.13, *p* < 0.01). Nest building score was affected by fluoxetine treatment in HS VEH compared to HS FLX (*p* < 0.01) while this was not seen in the LS VEH compared to LS FLX. Further, there was a difference between HS FLX compared to LS FLX and between HS VEH and LS VEH (Figure 3B).

Figure 3C shows that Kruskall Wallis test revealed differences in locomotor activity (*H*_(3,34)_ = 9.61, *p* < 0.05). Again, the effects of chronic fluoxetine were seen only in the HS mice (*p* < 0.01) and not in the LS mice. Further, there was no difference between LS VEH compared to LS FLX and between LS FLX compared HS FLX but there was a difference between LS FLX compared to HS FLX (*p* < 0.01).

Results of the splash test are shown on Figures 3D,E. Kruskall Wallis test revealed difference in the frequency of grooming (*H*_(3,34)_ = 11.01, *p* < 0.01) as well as in the duration of grooming (*H*_(3,34)_ = 13.99, *p* < 0.01). For both parameters, fluoxetine induced antidepressant-like effects both in HS and in LS mice. Indeed, concerning frequency of grooming, the Mann Whitney comparisons showed a significant difference between HS FLX compared to HS VEH (*p* < 0.01) and the same result was found for duration of grooming (HS VEH compared to HS FLX: *p* < 0.01). Further, there was a difference between LS VEH compared to LS FLX (*p* < 0.01 for the frequency and *p* < 0.05 for the duration of grooming). Finally, no significant differences were found between LS FLX compared to HS FLX and for HS VEH compared to LS VEH for duration and frequency of grooming.

Results obtained in the NSF test are shown on Figures 3F,G,H. Frequency of sniffing and latency to eat were modified by the treatment (respectively *H*_(3,34)_ = 14.05, *p* < 0.01 and *H*_(3,34)_ = 8.34, *p* < 0.01). This was related to the fact that fluoxetine elicited behavioral effects in both HS and LS animals. Indeed, Mann Whitney showed a significant difference between HS VEH and HS FLX for frequency of sniffing as well as latency to eat (*p* < 0.01). The HS and LS control groups only differed after fluoxetine treatment (for frequency of sniffing, *p* < 0.01). In addition, Kruskall Wallis *H*-test revealed difference in food consumption (*H*_(3,34)_ = 11.79, *p* < 0.01), which was related to a decreased consumption in the HS control group when compared to the corresponding fluoxetine condition (*p* < 0.01).

Results from the tail suspension test are presented in Figure 3I. Kruskal-Wallis *H*-test did not enable to reveal any statisticial difference among groups (*H*_(3,34)_ = 3.86, *p* = 0.276).

### Effects of UCMS and pharmacological treatments on HPA axis function

**Figure 4:**
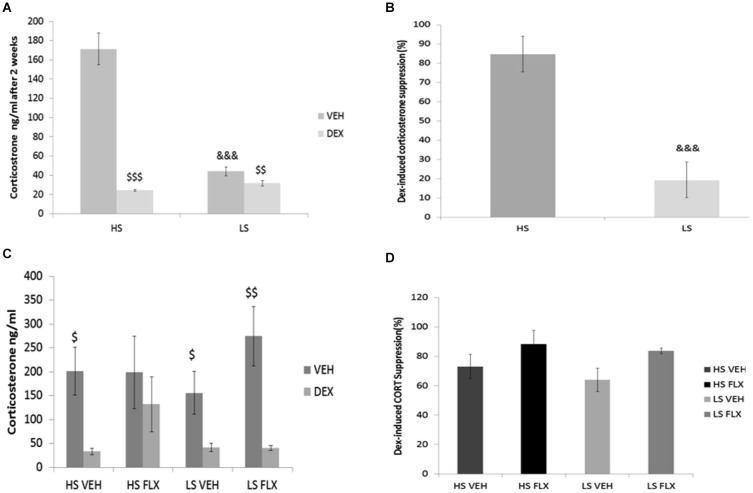
**Effects of UCMS and 4 week treatment with fluoxetine (15 mg/kg/day) on the dexamethasone (DEXA) suppression test after vehicle or dexamethasone injection**. HS: High suppressers, LS: Low suppressers, VEH: vehicle, FLX: fluoxetine. **(A)** Corticosterone after injection of vehicle or dexamethasone after 2 weeks of UCMS. **(B)** Suppression rate of corticosterone after 2 weeks of UCMS. **(C)** Corticosterone after injection of vehicle or dexamethasone at the end of UCMS. **(D)** Suppression rate after 9 weeks of UCMS. Figures 4A,B: *n* = 17 in each experimental condition. Figures 4C,D: HS VEH (*n* = 10), HS FLX (*n* = 7), LS VEH (*n* = 11), LS FLX (*n* = 6). HS vs. the corresponding LS condition: ^&&&^ *p* < 0.001; basal corticosterone vs. corresponding dexamethasone-induced corticosterone: ^$^ *p* < 0.05, ^$$^ *p* < 0.01, ^$$$^ *p* < 0.001.

Results indicate that after 2 weeks of drug-free UCMS, the basal corticosterone level was higher in the HS group compared to LS group (*p* < 0.001) (Figure 4A); this difference disappeared after dexamethasone. Further, at that time point, dexamethasone reduced the corticosterone level in both groups (Wilcoxon comparison: VEH vs. DEX for HS group *T* = 25, *P* < 0.001; VEH vs. DEX for LS group *T* = 0.00, *P* < 0.01). In addition, the dexamethasone suppression was different in the HS group compared to the LS group (*p* < 0.001) (Figure 4B).

At the end of the UCMS protocol, the four groups were not different statistically for basal corticosterone, corticosterone after dexamethasone (Figure 4C) and dexamethasone-induced corticosterone suppression *H*_(3,27)_ = 5.39, *p* > 0.05 (Figure 4D). Regarding the vehicle-dexamethasone comparisons in each experimental condition, Wilcoxon test revealed differences in all groups (*T* = 0.0, *P* < 0.05 for HS VEH batch, *T* = 0.00, *p* < 0.01 for LS VEH batch, *T* = 0.0, *p* < 0.05 for LS FLX batch) except for the HS FLX condition (*T* = 6, *p* > 0.5).

### Hippocampal cell profiferation and neurogenesis after pharmacolgical treatment

**Figure 5:**
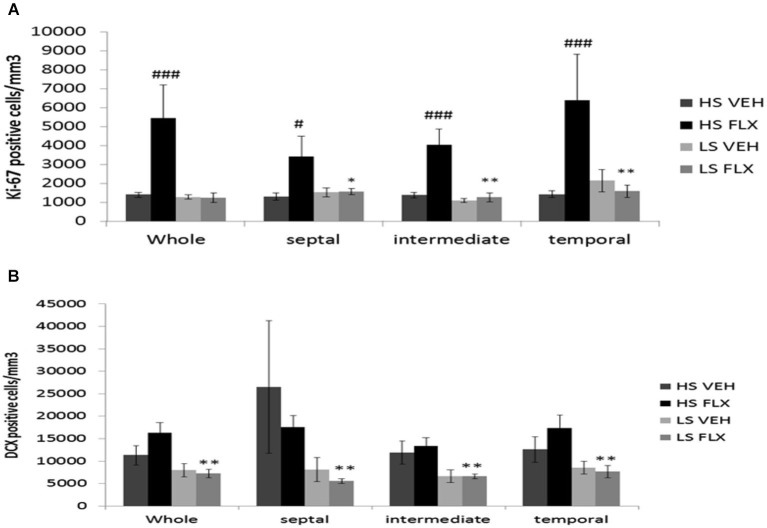
**Effects of UCMS and 4 weeks treatment of fluoxetine (FLX 15 mg/kg/day ip) on cell proliferation and neurogenesis in the hippocampus**. HS: High suppressers, LS: Low suppressers, VEH: vehicle, FLX: fluoxetine. **(A)** Densitiy of cell proliferation using ki-67 staining in the granule cell layer in the whole, dorsal, intermediate, and ventral hippocampus (whole, temporal and intermediate hippocampus: HS VEH (*n* = 9), HS FLX (*n* = 6), LS VEH (*n* = 7), LS FLX (*n* = 6); septal hippocampus: HS VEH (*n* = 7), HS FLX (*n* = 6), LS VEH (*n* = 7), LS FLX (*n* = 5)). **(B)** Density of immature neurons using doublecortin (DCX) labeling in the GCL (whole, temporal and intermediate parts: HS VEH (*n* = 5), HS FLX (*n* = 6), LS VEH (*n* = 6), LS FLX (*n* = 4); septal parts: HS VEH (*n* = 5), HS FLX (*n* = 5), LS VEH (*n* = 6), LS FLX (*n* = 4)). HS VEH vs. HS FLX: ^#^ *p* < 0.05, ^###^ *p* < 0.001; HS FLX vs. LS FLX: * *p* < 0.05, ** *p* < 0.01.

As seen from Figure 5A, Kruskall Wallis *H*-test revealed a significant difference in cell proliferation among groups in the different regions of the granule cell layer of the dentate gyrus (whole dentate gyrus: *H*_(3, 28)_ = 13.84; *p* < 0.01; septal dentate gyrus: *H*_(3,23)_ = 7.46; *p* < 0.05; intermediate dentate gyrus: *H*_(3,28)_ = 14.04; *p* < 0.01 and temporal dentate gyrus: *H*_(3,28)_ = 12.47; *p* < 0.01). Overall, fluoxetine induced a marked increase of the density of ki-67 positive cells only in the HS subgroup. Mann Whitney showed significant difference between HS VEH vs. HS FLX in the whole of dentate gyrus (*p* < 0.001), the septal region (*p* < 0.05), the intermediate region (*p* < 0.001) and the temporal region of the dentate gyrus (*p* < 0.001). No effect of fluoxetine was seen in the LS group in the different parts of the hippocampus. HS and LS markedly differed only after fluoxetine.

Concerning doublecortin positive cells (see Figure 5B), Kruskall Wallis *H*-test revealed significant differences in all regions of the hippocampus (whole dentate gyrus: *H*_(3,21)_ = 10.37, *p* < 0.05; septal part: *H*_(3,20)_ = 9.13; *p* < 0.05; intermediate part *H*_(3,20)_ = 1.49, *p* < 0.01; temporal part: *H*_(3,21)_ = 7.91; *p* < 0.05). No effects of fluoxetine were observed in HS or LS mice. Further, there was no difference between HS VEH and HS FLX. Finally, there was a difference between HS FLX and LS FLX in the whole (*p* < 0.01), the septal (*p* < 0.01), the intermediate (*p* < 0.01) and the temporal region *p* < 0.01, the DCX labeling being lower in the HS mice.

**Figure 6:**
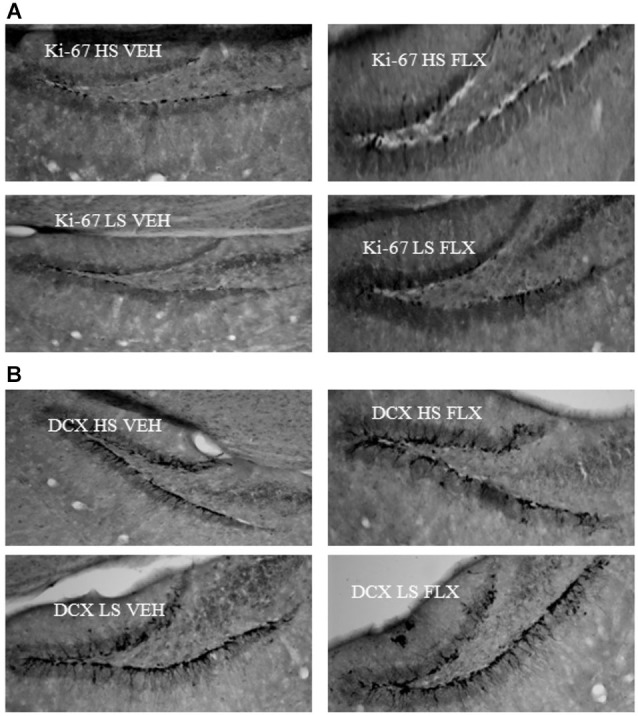
**Representative pictures of ki67 (6A) and DCX (6B) labeling in the dentate gyrus of the hippocampus**. HS: High suppressers, LS: Low suppressers, VEH: vehicle, FLX: fluoxetine.

Representative pictures of ki-67 and DCX labeling can be seen from Figure 6.

## Discussion

In this experiment we studied the contribution of HPA axis dysregulation in creating resistance to antidepressants using an animal model of major depression. We tested the effect of fluoxetine in two groups based on the suppression of corticosterone following dexamethasone administration after an initial period of stress. Our results demonstrate that fluoxetine-induced antidepressant effects were observed with higher amplitude in a group initially characterized as HS when compared to a group initially characterized as LS in various behavioral tests such as the nest test, the actograph and the novelty suppression of feeding. This absence of antidepressant-like effects of fluoxetine in the LS group mimicked the antidepressant resistance seen in humans in whom failure of SSRI to induce remission has been observed in correlation with a defect of the regulation of the HPA axis. We can therefore propose that mice from the LS group can be considered as a way to induce HPA-related antidepressant resistance. It is however important to indicate that this resistance observed in the LS group does not concern all aspects of the phenotype. Indeed, the effects of fluoxetine were still present on the coat state (even if the amplitude of the effects is lower) and in the splash test, two phenotypes related to grooming behavior. This suggests that there is a clear dissociation between the mechanisms underlying the effects of fluoxetine on grooming-related behavior and the mechanisms underlying the other behavioral actions of the SSRI. Phenotyping the UCMS-induced anhedonia would be necessary to enable to further characterize the effects of the SSRI in HS and LS mice. Further, it is interesting to note that the behavior of mice from the vehicle-treated HS and the vehicle-treated LS groups did not differ after vehicle treatment, indicating that the contribution of the HPA regulation is specific to the antidepressant’s effects.

Further, we found that chronic fluoxetine treatment was able to increase cell proliferation in the dentate gyrus of the hippocampus in mice from the HS group. This result is in agreement with previous findings (Santarelli et al., 2003; Airan et al., 2007; Wang et al., 2008). This effect was observed at all levels of the dentate gyrus, from the more septal to the more temporal ones. Interestingly, in mice from the LS group, fluoxetine was unable to increase cell proliferation. Therefore, the inability of fluoxetine to induce behavioral effects in mice from the LS group correlated with a failure to increase cell proliferation in the hippocampus, suggesting a possible mechanism underlying the antidepressant-resistance. However, it is important here to consider that, as indicated above, fluoxetine was able to modify grooming and coat state in mice from the LS group, suggesting that these two effects are independent of hippocampal cell proliferation. This confirmed the dissociation between neurogenesis-dependent and independent effects, which have been observed previously by our group (Surget et al., 2008) and others (Bessa et al., 2009; David et al., 2009; Mateus-Pinheiro et al., 2013). Interestingly also, the level of cell proliferation was unrelated to the basal corticosterone levels, as the increase in cell proliferation observed in the HS fluoxetine group was not associated to a decreased basal corticosterone.

In the present study, fluoxetine was unable to alter doublecortin labeling in mice from both the LS and HS groups. This finding has already been observed by others (see for example David et al., 2010) and might be related to the fact that fluoxetine accelerates the maturation of the newborn cells. Indeed, others showed that fluoxetine does not increase the number of doublecortin positive cells, but increases the fraction of doublecortin-positive cells possessing tertiary dendrites (David et al., 2010).

Concerning basal corticosterone and dexamethasone suppression test, we segregated the mice according to the dexamethasone suppression level observed after 2 weeks of stress. Interestingly, this fully overlapped with the basal corticosterone values observed at this time point, as HS overlaps with high basal corticosterone. Thus it is possible that the absence of suppression in mice from the LS group could be related to a ceiling effect, as it might be difficult to further reduce the corticosterone level in this group. Further, the high basal corticosterone exhibited by mice of the HS group could relate either to an initial difference present before the beginning of the UCMS, or to an increased vulnerability to stress in mice from this group. The data concerning corticosterone levels and dexamethasone suppression at the end of the 9 weeks of UCMS can be interpreted within this frame. Indeed, the basal corticosterone levels observed in control mice of the LS group at the end of the UCMS are equivalent to the ones of the mice from the HS group after 2 weeks of UCMS. Therefore, it is possible that the elevated corticosterone level observed in mice of the HS group after 2 weeks of UCMS could not be further increased. At the end of the UCMS, dexamethasone could induce corticosterone suppression due to the high basal levels of corticosterone present in all groups.

How can we explain that fluoxetine induced some behavioral effects and an action on cell proliferation in the HS, but not in the LS group? We think that the inability of fluoxetine to increase cell proliferation in mice of the LS group might certainly explain the failure of the antidepressant to induce behavioral effects. In fact, we propose that in HR mice, the high level of corticosterone observed after 2 weeks of UCMS might have induced a decrease in hippocampal glucocorticoid receptors (GRs), both in adult granule and progenitor cells. This downregulation of GRs in the progenitor cells might then have accelerated the maturation of newborn neurons, as such an effect has already been observed (Fitzsimons et al., 2013), eliciting the behavioral effects. This effect of corticosterone would not be present in the mice from the LS group displaying low corticosterone after 2 weeks of UCMS. However, this is purely speculative and further assays, such as quantification of GRs in our two groups, should confirm the present interpretation.

In conclusion, these data suggest that dysregulation of the HPA axis is associated with antidepressant resistance. Even if the data are only correlative, they suggest that this fluoxetine non-response is related to poor ability of the SSRI to stimulate cell proliferation in the dentate gyrus. Further studies are necessary to investigate the causal relationship between these phenomenons.

## Conflict of interest statement

The authors declare that the research was conducted in the absence of any commercial or financial relationships that could be construed as a potential conflict of interest.
